# The Activin Branch Ligand Daw Regulates the *Drosophila melanogaster* Immune Response and Lipid Metabolism against the *Heterorhabditis bacteriophora* Serine Carboxypeptidase

**DOI:** 10.3390/ijms25147970

**Published:** 2024-07-21

**Authors:** Sreeradha Mallick, Eric Kenney, Ioannis Eleftherianos

**Affiliations:** Infection and Innate Immunity Lab, Department of Biological Sciences, The George Washington University, Washington, DC 20052, USA; sreeradha.mallick@gwmail.gwu.edu (S.M.); etkenney2@gwmail.gwu.edu (E.K.)

**Keywords:** *Drosophila*, *Heterorhabditis*, serine carboxypeptidase, virulence factor, innate immunity, immune gene expression, lipid metabolism

## Abstract

Despite impressive advances in the broad field of innate immunity, our understanding of the molecules and signaling pathways that control the host immune response to nematode infection remains incomplete. We have shown recently that Transforming Growth Factor-β (TGF-β) signaling in the fruit fly *Drosophila melanogaster* is activated by nematode infection and certain TGF-β superfamily members regulate the *D. melanogaster* anti-nematode immune response. Here, we investigate the effect of an entomopathogenic nematode infection factor on host TGF-β pathway regulation and immune function. We find that *Heterorhabditis bacteriophora* serine carboxypeptidase activates the Activin branch in *D. melanogaster* adults and the immune deficiency pathway in Activin-deficient flies, it affects hemocyte numbers and survival in flies deficient for Activin signaling, and causes increased intestinal steatosis in Activin-deficient flies. Thus, insights into the *D. melanogaster* signaling pathways and metabolic processes interacting with *H. bacteriophora* pathogenicity factors will be applicable to entomopathogenic nematode infection of important agricultural insect pests and vectors of disease.

## 1. Introduction

Insects have developed morphological, behavioral, and physiological defenses to combat parasitic nematode infection [1,2]. Most studies have focused on the immune response of natural hosts against entomopathogenic nematodes (EPNs) and the immune response of mosquitoes and black flies against filarial nematodes [3,4,5]. Insects activate both humoral and cellular immune responses to nematode infection as well as the phenoloxidase (PO) and coagulation cascades that lead to melanotic encapsulation [6,7,8,9,10,11,12,13,14,15,16,17]. Some nematode parasites have evolved strategies to evade or suppress the insect immune system by preventing or disrupting the activation of immune responses to promote their survival in the host [18,19,20,21,22,23,24,25,26].

Previous work has demonstrated the power of using the fruit fly model *Drosophila melanogaster* for dissecting the molecular/genetic basis of insect anti-nematode immune response [27,28,29,30,31,32,33,34]. Infection of *D. melanogaster* larvae with *Heterorhabditis bacteriophora* EPNs upregulates four antimicrobial peptide genes [27]. The antimicrobial peptide response is specific to their *Photorhabdus luminescens* mutualistic bacteria because axenic nematodes (those lacking their associated bacteria) fail to induce antimicrobial peptide genes. Also, we have found that *H. bacteriophora* nematodes upregulate several immune-related genes in adult *D. melanogaster*, but injection of *P. luminescens* bacteria alone results in lower levels of gene expression in the flies [30]. Inactivation of *D. melanogaster* transglutaminase, a conserved component of clotting cascades in insects and humans, results in decreased aggregation of zymosan beads and increased sensitivity of larvae to *H. bacteriophora* infection [33]. Of note, the clotting factors gp150 and fondue participate in the *D. melanogaster* anti-nematode response [33], and a homolog of thioester-containing complement protein 3, a basement membrane component (glutactin), a recognition protein (GNBP-like 3) and several small peptides contribute to the control of *H. bacteriophora* infection in *D. melanogaster* larvae [31]. Recently, we have identified differentially regulated genes in *H. bacteriophora* that are potentially involved in the process of infection and therefore are expected to interfere with *D. melanogaster* immune processes [35]. 

Our recent work has further demonstrated the participation of TGF-beta signaling in the *D. melanogaster* immune response against EPN infection and wounding [36]. More precisely, we have recently revealed a novel role for the TGF-β signaling pathway in the fly anti-nematode immunometabolic response [37]. We have shown that inactivation of *Daw* or *Dpp* regulates the survival of *D. melanogaster* flies to infection by two *Heterorhabditis* nematode species and their mutualistic bacteria whereas inactivation of *Daw* reduces nematode persistence in the mutant flies [38]. Also, the inactivation of *Mad* or *Dpp* promotes fly survival and increases antimicrobial peptide gene expression levels upon sterile injury or nematode infection, respectively, but not upon bacterial challenge [39]. Furthermore, extracellular ligand *Scw* and Type I receptor *Sax* in the BMP pathway as well as the Type I receptor *Babo* in the Activin pathway are substantially upregulated following *Heterorhabditis gerrardi* infection, which leads to activation of the intracellular component *Mad* [40]. Finally, we have demonstrated that crosstalk between TGF-β signaling and NF-κB immune signaling occurs at the extracellular level and is specific to *H. gerrardi* infection [41]. 

In the current work, we expand these findings by connecting the *D. melanogaster* TGF-β signaling regulation and immunometabolic function with *H. bacteriophora* nematode infection factor serine carboxypeptidase. This information will help us understand the molecular interplay between insect immune signaling and parasitic nematode effectors that promote infection.

## 2. Results

### 2.1. H. bacteriophora Recombinant Serine Carboxypeptidase (rSCP) Induces Activin and Imd Signaling Activity in Drosophila

**Figure 1:**
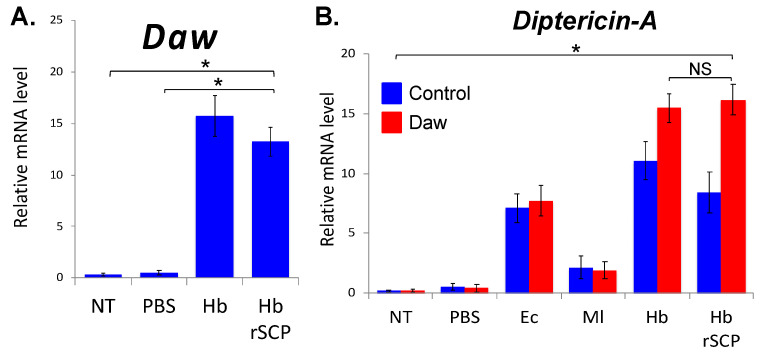
*Heterorhabditis bacteriophora* recombinant serine carboxypeptidase (rSCP) activates Activin and Imd signaling in *Drosophila melanogaster*. Injection of *H. bacteriophora* (Hb) rSCP into: (**A**). *Daw* (Activin pathway) is upregulated in w^1118^ background control flies (ANOVA, *F* = 1.222, * *p* < 0.05), (**B**). *Diptericin-A* (Imd pathway) is upregulated in *Daw* mutants (ANOVA, *F* = 1.137, * *p* < 0.05). Each experiment was repeated three times and each experimental condition involved approximately 30 adult (5–10 days old) flies. NT: Non-Treated flies; Time-point: 24 h; NS: Non-Significant. Error bars represent standard errors.

To examine whether certain *H. bacteriophora* secreted proteins modulate the signaling capacity in the fly, we have injected 7 ng in 69 nl of *H. bacteriophora* rSCP (it corresponds to the amount produced by approx. 100 *H. bacteriophora* axenic nematodes during infection, unpublished data) into *w*^1118^ wild-type flies (Control) and used qRT-PCR and gene-specific primers to test the activation levels of TGF-β and Imd signaling in adult *D. melanogaster*. We assessed Imd signaling activity because *H. bacteriophora* produces molecules that facilitate infection through suppression of this pathway [42]. We have found that the Activin extracellular ligand *Daw* is significantly upregulated at 24 h post-injection with *H. bacteriophora* rSCP (Figure 1A). As before, we have found that *H. bacteriophora* infection upregulates *Daw* in *D. melanogaster* adults [38]. We have further shown that injection of *H. bacteriophora* rSCP significantly upregulates the expression of the antimicrobial peptide gene *Diptericin-A* in *Daw* loss-of-function mutant flies (line Pbac{XP}daw^05680^) compared to their background controls and the other treatments (Figure 1B). These results indicate that *H. bacteriophora* rSCP regulates TGF-β signaling in *D. melanogaster* and in the absence of the Activin branch also modulates innate immune signaling in the fly.

### 2.2. H. bacteriophora Recombinant Serine Carboxypeptidase Alters the Cellular Immunity and Survival Ability of Activin-Deficient Drosophila

**Figure 2:**
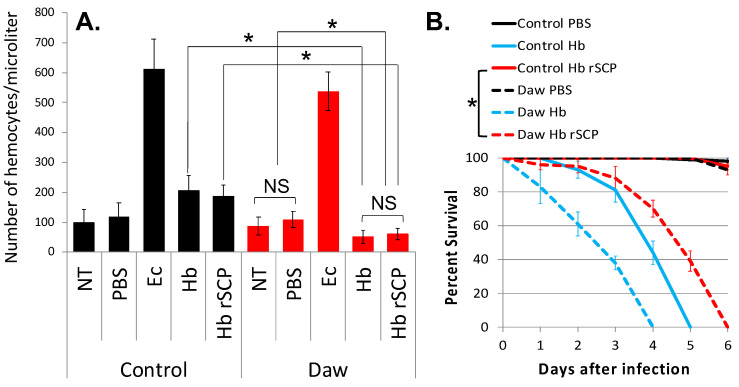
Effect of *Heterorhabditis bacteriophora* recombinant serine carboxypeptidase (rSCP) on hemocyte numbers and survival of *Drosophila melanogaster* Activin deficient flies. Injection of purified rSCP from *H. bacteriophora* (Hb) nematodes into *Daw* mutant flies reduces (**A**). hemocyte numbers (24 h) (ANOVA, *F* = 1.115, * *p* < 0.05) and (**B**). survival ability compared to the w^1118^ background flies (Control). Each experiment was replicated three times with 90 adult (5–10 days old) flies per experimental condition. (Mantel-Cox, *df* = 1, * *p* < 0.05); NS: Non-Significant. Error bars represent standard errors.

To explore the participation of TGF-β signaling in the cellular immune response and survival ability of *D. melanogaster* in response to entomopathogenic nematode infection factors, we have injected 7 ng of *H. bacteriophora* rSCP into *w*^1118^ flies (Control) and *Daw* loss-of-function mutant flies (line Pbac{XP}daw^05680^), and 24 h later we have recorded numbers of circulating hemocytes. We have counted substantially fewer hemocytes in *Daw* mutants compared to *w*^1118^ controls and compared to *Daw* mutants injected with PBS or non-treated individuals (Figure 2A). We also estimated the survival response of the two *D. melanogaster* lines to injection with *H. bacteriophora* rSCP and found that background control flies are able to survive the challenge, whereas *Daw* mutant flies succumb at 6 days post injection (Figure 2B). Also, as we have shown before [38], *Daw* mutant flies are more sensitive to *H. bacteriophora* nematode infection compared to background controls. These findings indicate that *H. bacteriophora* rSCP confers pathogenicity to *D. melanogaster* in the absence of Activin signaling, and *Daw* can regulate the hemocyte population in the adult fly during response to an entomopathogenic nematode infection factor.

### 2.3. H. bacteriophora Infection of Activin-Deficient Drosophila Flies Results in Perturbed Intestinal Lipid Homeostasis

**Figure 3:**
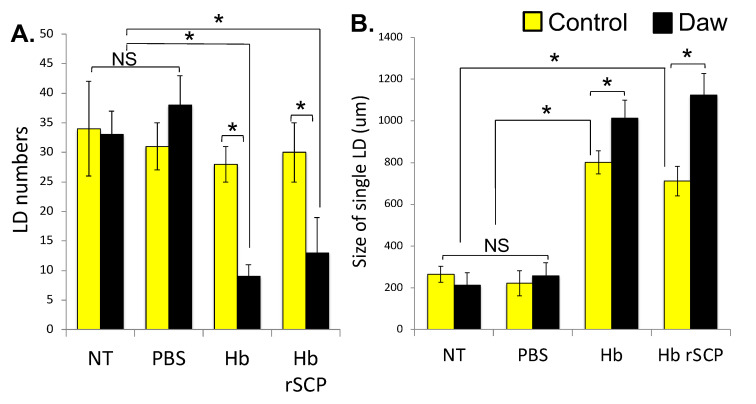
Increased intestinal steatosis in Activin-deficient *Drosophila melanogaster*. (**A**). Lipid droplet (LD) numbers decrease (ANOVA, *F* = 1.301, * *p* < 0.05), and (**B**). LD size increases (ANOVA, *F* = 1.107, * *p* < 0.05) in the gut of *Daw* mutant flies infected with *Heterorhabditis bacteriophora* (Hb) nematodes or injected with Hb recombinant serine carboxypeptidase (rSCP). The experiment was performed three times with each experimental treatment including in total 90 adult (5–10 days old) flies. NS: Non-Significant. Error bars represent standard errors.

We have shown recently that intestinal lipid droplets in *D. melanogaster* mediate the antibacterial response [43]; therefore, here we first examined whether lipid droplets can also regulate anti-nematode immunity in the fly. For this, we infected single *w*^1118^ flies with approximately 100 *H. bacteriophora* axenic infective juveniles, or we injected *w*^1118^ individuals with rSCP from *H. bacteriophora* axenic nematodes and examined changes in numbers and size of lipid droplets 24 h later. No treatment or injection with PBS served as negative controls. We have found that *H. bacteriophora*-infected flies or those injected with *H. bacteriophora* rSCP have significantly reduced accumulation of lipid droplets in the gut and increased lipid droplet size as compared to the PBS-injected and non-treated individuals (Figure 3A and Figure 3B, respectively). In addition, this lipid droplet phenotype in the fly gut is exacerbated in *Daw* loss-of-function mutants (line Pbac{XP}daw^05680^) compared to *w*^1118^ background controls (Figure 3A,B). Thus, the Activin branch of the TGF-β signaling pathway in *D. melanogaster* regulates intestinal lipid homeostasis in response to entomopathogenic nematode infection or challenge with an entomopathogenic nematode infection factor.

## 3. Discussion

Our results reveal that *Daw* is substantially upregulated in wild-type flies upon injection of *H. bacteriophora* rSCP, and because it is a secreted signal [44], it may function systemically to regulate the expression of downstream genes in the Activin branch of TGF-β signaling. Also, because *H. bacteriophora* infection or injection of *H. bacteriophora* rSCP substantially upregulates *Diptericin-A* expression in *Daw* loss-of-function mutant flies, we postulate that certain entomopathogenic nematode infection factors, such as the *H. bacteriophora* SCP, are capable of altering the immune signaling in *D. melanogaster* when the Activin branch is inactivated. 

The current data also suggest that Activin-deficient flies are sensitive to injection with the *H. bacteriophora* rSCP infection factor, and this phenotype is accompanied by increased Imd signaling activity and reduced hemocyte numbers. These findings strongly indicate a close interaction between this entomopathogenic nematode infection factor and the regulation of immune signaling and function in *D. melanogaster* deficient for the TGF-β Activin branch. Therefore, we speculate that the Activin pathway in the fly regulates cellular and humoral immune processes against challenge with the *H. bacteriophora* SCP. 

Our results further indicate that infection of *D. melanogaster* wild-type flies with *H. bacteriophora* nematodes or injection with *H. bacteriophora* rSCP results in perturbed intestinal lipid metabolism marked by intestinal steatosis. These findings point out a relationship between Activin signaling activity and lipid metabolism in the context of entomopathogenic nematode infection.

Future work will study the conservation of these processes by expanding to other entomopathogenic nematodes. Our recent work indicates that excreted-secreted products from *Steinernema carpocapsae* manipulate the *D. melanogaster* immune response and they may also affect TGF-β signaling [45]. In future studies, we will perform time-course experiments to elucidate the causal relationships between Activin signaling activity and regulation of hemocyte numbers and lipid metabolism, which will provide a more complete picture on the dynamics of TGF-β signaling activation and immune responses. More precisely, we will focus on analyzing the tissue-specific transcript levels of TGF-β signaling molecules and exploring the immune signaling activity in various tissues of wild-type flies as well as TGF-β signaling mutant flies upon injection with the *H. bacteriophora* SCP infection factor. Another possibility will be to explore the involvement of other Activin branch members in cellular immune reactions of *D. melanogaster* against the *H. bacteriophora* SCP and identify whether (and how) cellular immune reactions in Activin mutant flies interfere or coordinate with humoral immunity. Also, investigating potential feedback loops between immune activation and metabolic changes in the context of entomopathogenic nematode infection factors may reveal interesting host-parasite dynamics. Finally, it will be interesting to investigate whether inactivating the Activin branch in *H. bacteriophora*-infected flies or flies injected with the *H. bacteriophora* SCP leads to changes in lipogenesis regulation in various fly tissues.

The fruit fly *D. melanogaster* is able to mount rapid and efficient reactions in response to diverse pathogens, including entomopathogenic nematodes [46]. Many of these anti-pathogen immune responses are remarkably conserved across insect species. The study of *D. melanogaster* is highly relevant to other Diptera, such as mosquitoes and leaf miners, given the evolutionary conservation of many of the key signal transduction pathways and transcriptional regulators that control innate immunity [47]. Deciphering the molecular and functional basis of the *D. melanogaster* immune response against insect parasitic nematodes and the infection factors they produce during the different stages of infection will provide a better understanding on the mechanisms that have evolved to oppose nematode attacks [2,48]. This information will in turn allow us to design alternative approaches to tackle agricultural insect pests and disease vectors effectively.

## 4. Materials and Methods

### 4.1. Fly Stocks

*Drosophila melanogaster* stocks were maintained and amplified at 25 °C and a 12:12-h light:dark cycle on Bloomington Drosophila Stock Center cornmeal food (Labexpress) supplemented with yeast (Carolina Biological Supply, Burlington, NC, USA). Adult flies (5–10 days old) carrying P-bac insertion Pbac{XP}daw^05680^ (strain d05680, Exelixis, Boston, MA, USA) and background control line *w*^1118^ (strain 3605, Bloomington, IL, USA) were used in all experiments. Both fly strains have been used consistently in our previous research [37,38,49]. 

### 4.2. Nematode and Bacterial Stocks

*H. bacteriophora* strain TT01 infective juveniles were kept in tissue culture flasks and multiplied through *Galleria mellonella* larvae that were placed on water traps [50]. This method involved the infection of *G. mellonella* larvae (kept in Petri dishes) with nematode infective juveniles (kept in tissue culture flasks) at 25 °C, the preparation of water traps with folded filter paper, the placement of the dead *G. mellonella* containing infective juveniles in the water trap 12 days after nematode infection, the migration of the new generation of infective juveniles from the dead insects to the water, and the storage of the new infective juveniles into tissue culture flasks [51]. One- to four-week-old nematodes were used in the experiments. Axenic nematodes without their symbiotic *P. luminescens* bacteria were generated using a previously developed method, where infective juveniles were amplified in fifth or sixth instar *G. mellonella* caterpillars that had been previously infected with the RET16 derivative of *Photorhabdus temperata* strain NC1 [42]. Bacterial cultures involved *Escherichia coli* (strain DH5a) and *Micrococcus luteus* (strain CIP A270). The bacteria were cultured on petri dishes containing 2.5% Luria-Bertani (LB) and 1.5% agar (Difco Laboratories, Detroit, MI, USA). Bacterial liquid cultures were prepared in sterile tubes containing 10 mL of 2.5% LB and incubated for 24 h on a rotary shaker at 265 rpm at 37 °C for *E. coli* and at 30 °C for *M. luteus*. Bacterial cultures were centrifuged at 4 °C and resuspended in phosphate-buffered saline (PBS) before their optical density (600 nm) was determined with a spectrophotometer (NanoDrop 2000c; Thermo Fisher Scientific, Waltham, MA, USA).

### 4.3. Production of Nematode Recombinant Serine Carboxypeptidase

The protocol for production and purification of recombinant *H. bacteriophora* serine carboxypeptidase has been described in detail before [52].

### 4.4. Fly Infections

*Drosophila melanogaster Daw* mutants and *w*^1118^ adult flies were infected with 100 infective juveniles of *H. bacteriophora* suspended in 250 μL of sterile water. Nematode suspensions were added to plastic vials containing four filter papers (Whatman Grade 1, 20 mm) at the bottom and 250 μL of 1% sucrose. Uninfected control flies were kept in vials containing sterile water and 1% sucrose only. All vials were covered with plugs, which were lowered close to the bottom of the vials to bring flies and nematodes in close proximity and facilitate infection. Fly infections with bacteria were performed by delivering 18.4 nl of a PBS suspension containing approximately 500 cells of *E. coli* or *M. luteus* into the hemocoel of *D. melanogaster* adults at the lateral anterior part of the thorax using a Nanoject II apparatus (Drummond Scientific, Broomall, PA, USA). Injections with PBS alone served as negative controls. All infected and control flies were kept in vials containing cornmeal food at 25 °C and a 12:12-h light:dark cycle. Each replicate included five male and five female 5–10 days old adult flies. Each infection was repeated three times with three replicates per treatment and different batches of flies, nematodes, and bacteria. Fly survival was estimated daily and up to six days post-infection.

### 4.5. Gene Expression Analysis

RNA was isolated from 4–5 *D. melanogaster Daw* mutants and *w*^1118^ adult flies (5–10 days old) using the Trizol reagent (Life Technologies, Frederick MD, USA) protocol. Reverse transcription was carried out following the instructions in the High-Capacity cDNA Reverse Transcription Kit (Applied Biosystems, Foster City, CA, USA). Real time PCR assays were performed in a CFX96 Real-Time System, C1000 Thermal Cycler (Bio-Rad, Philadelphia, PA, USA) using the GreenLink qPCR Mix (BioLink, Cary, NC, USA) and the following primers: *Daw* (CG16987) Forward GGTGGATCAGCAGAAGGACT, *Daw* Reverse GCCACTGATCCAGTGTTTGA, *Diptericin-A* (CG12763) Forward GCTGCGCAATCGCTTCTACT, *Diptericin-A* Reverse TGGTGGAGTTGGGCTTCATG, *RpL32* (CG7939) Forward GATGACCATCCGCCCAGCA, *RpL32* Reverse CGGACCGACAGCTGCTTGGC. The cycling conditions were 95 °C for 2 min, 40 repetitions of 95 °C for 15 s followed by 61 °C for 30 s, and then one round of 95 °C for 15 s, 65 °C for 5 s, and finally 95 °C for 5 s. Gene expression from the RT-qPCR experiments was analyzed in accordance with the 2^−ΔΔCT^ method [53,54]. Each experiment involved biological duplicates and three technical replicates per sample and was repeated three times with different batches of flies and nematodes.

### 4.6. Hemocyte Count Estimation

Hemolymph was extracted from *D. melanogaster Daw* mutants and *w*^1118^ adult flies 24 h after infection with *H. bacteriophora* axenic nematodes or injection with rSCP, *E. coli*, or PBS. Untreated flies were used as negative controls. For hemocyte count estimation, hemolymph from 10 flies was first collected in 30 μL of 2.5× protease inhibitor cocktail (Sigma-Aldrich, Burlington, MA, USA), hemolymph samples were pipetted on a hemocytometer, and hemocyte numbers were recorded on a compound microscope (Olympus CX21, Center Valley, PA, USA) at 40× magnification. Fly hemocyte counting experiments were run three times with different batches of flies and nematodes, bacteria, and each experiment involved biological and technical triplicates.

### 4.7. Lipid Droplet Count and Size Estimation

*Drosophila melanogaster Daw* mutants and *w*^1118^ 5–10-day old adult flies were infected with *H. bacteriophora* axenic nematodes or injected with rSCP or PBS, and 24 h later their gut tissues were dissected, stained in Nile red, and mounted in ProLong™ Diamond AntiFade Mountant with DAPI (Life Technologies) before imaging and lipid droplet counting on a Zeiss (White Plains, NY, USA) LSM 510 confocal microscope, as previously described [37]. Lipid droplet size quantification was carried out using ImageJ software 1.54e (National Institutes of Health). The experiment was replicated three times with different batches of flies and nematodes, and each experiment involved biological and technical triplicates. 

### 4.8. Statistical Analysis

GraphPad Prism 8 was used for statistical analysis of the data and construction of the figures. Results from the survival experiments were analyzed using the log-rank (Mantel-Cox) test. Results from the rest of the experiments were analyzed using one-way analysis of variance (ANOVA) and Tukey *post-hoc* tests. A *p*-value < 0.05 was considered statistically significant.

## Data Availability

The data supporting the findings of this study are available within the article.
